# Mammal traits and soil biogeochemistry: Functional diversity relates to composition of soil organic matter

**DOI:** 10.1002/ece3.10392

**Published:** 2023-08-17

**Authors:** María Losada, Mar Sobral, Kirsten M. Silvius, Sara Varela, Antonio M. Martínez Cortizas, José M. V. Fragoso

**Affiliations:** ^1^ EcoPast (GI‐1553), Departmento de Edafoloxía e Química Agrícola, Facultade de Bioloxía Universidade de Santiago de Compostela Santiago de Compostela Spain; ^2^ Department of Forest Resources and Environmental Conservation Virginia Tech Blacksburg Virginia USA; ^3^ MAPAS Lab, Departamento de Ecoloxía e Bioloxía Animal Universidade de Vigo Vigo Spain; ^4^ CRETUS – EcoPast (GI‐1553), Departmento de Edafoloxía e Química Agrícola, Facultade de Bioloxía Universidade de Santiago de Compostela Santiago de Compostela Spain; ^5^ Departamento de Zoologia Universidade de Brasília Brasília Brazil; ^6^ Institute of Biodiversity Science and Sustainability California Academy of Sciences San Francisco California USA

**Keywords:** biogeochemical cycles, ecosystem functioning, functional diversity, mammal traits, soil organic matter composition, SOM recalcitrance, tropical soils

## Abstract

Mammal diversity affects carbon concentration in Amazonian soils. It is known that some species traits determine carbon accumulation in organisms (e.g., size and longevity), and are also related to feeding strategies, thus linking species traits to the type of organic remains that are incorporated into the soil. Trait diversity in mammal assemblages – that is, its functional diversity – may therefore constitute another mechanism linking biodiversity to soil organic matter (SOM) accumulation. To address this hypothesis, we analyzed across 83 mammal assemblages in the Amazon biome (Guyana), the elemental (by ED‐XRF and CNH analysis) and molecular (FTIR‐ATR) composition of SOM of topsoils (401 samples) and trait diversity (functional richness, evenness, and divergence) for each mammal assemblage. Lower mammal functional richness but higher functional divergence were related to higher content of carbonyl and aliphatic SOM, potentially affecting SOM recalcitrance. Our results might allow the design of biodiversity management plans that consider the effect of mammal traits on carbon sequestration and accumulation in soils.

## Graphical abstract

Mammal diversity affects carbon concentration in soils, but the diversity of traits in mammal assemblages – that is, its functional diversity – may constitute another mechanism linking biodiversity to soil organic matter (SOM) accumulation. We found that lower mammal functional richness but higher functional divergence increases the content of carbonyl and aliphatic SOM, potentially affecting SOM recalcitrance. Our results might allow the design of biodiversity management plans that consider the effect of mammal traits on carbon sequestration and accumulation in soils.

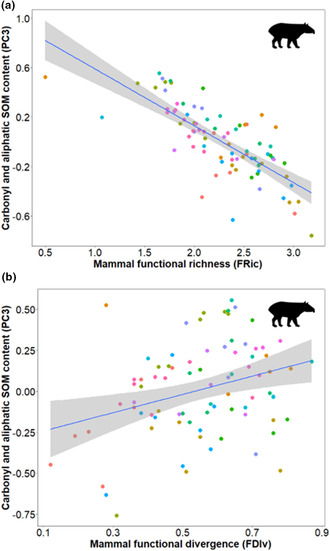

## 1 INTRODUCTION

Functional diversity describes the variation of traits of species coexisting in an ecological community (Petchey & Gaston, 2006), and is linked to ecosystem services (Krause et al., 2014). Some species traits are directly related to the amount of carbon accumulated in organisms (e.g. body size) or to accumulation time (e.g. longevity; Luyssaert et al., 2008), while others are related to the type of feeding interactions between species (e.g. herbivory, carnivory, frugivory) which are linked to carbon fluxes in the ecosystem (Ayres et al., 2010; Forbes et al., 2019; Schmitz & Leroux, 2020). In a previous investigation, we showed that mammal diversity not only affects carbon concentration in soils (Sobral et al., 2017), but that also can be linked to the composition of soil organic matter—SOM (Losada et al., 2023).

Carbon economics shapes trait combinations that species can display (Blumstein et al., 2022; Castorena et al., 2022). In fact, combinations of traits are associated on the one hand with life‐history strategies, from slow to fast life histories, and on the other, with resource use, from conservative to acquisitive strategies (Brown et al., 2018; Capdevila et al., 2020; Robert Burger et al., 2021). Both dimensions are related to the reserves and flows of matter and energy—mainly carbon—of ecosystems, such as biomass and productivity (Enquist et al., 2020; Hatton et al., 2015, 2019). Mammal species differ in body size, weight, fecundity, food preferences, and daily activity (Flynn et al., 2009; Gorczynski & Beaudrot, 2021; Hempson et al., 2015). Thus, the diversity of traits within mammal assemblages, i.e., functional diversity, may constitute another mechanism that would relate species diversity to carbon cycling (Sobral et al., 2023).

Variation in traits between species (e.g., between herbivores, carnivores, omnivores, and insectivores) influences the type of trophic interactions and the type of organic remains animals produce through their feeding activities (Andriuzzi & Wall, 2018; Cromsigt et al., 2018; Kristensen et al., 2022). Thus, the trophic level of the species will be related to the content and type of composition of the organic remains incorporated into the soil. The excrements of carnivorous animals have a lower C:N ratio than those of frugivorous or herbivorous animals. Furthermore, the C:N ratio of herbivore dung is similar to that of leaves, whereas that of carnivores is much lower and optimal for microbial growth (Benbow et al., 2019). Large mammal species are key components of the tropical forest fauna, including felid and canid predators, frugivorous primates and ungulates, folivores and herbivores, among others (Fragoso et al., 2016, 2019). Collectively, the organic remains generated by these mammal assemblages alter carbon inputs to the soil and affect the molecular composition of SOM.

Molecular composition of SOM determines its resistance to microbial decomposition, with carbohydrates being the simplest and labile, aromatic and aliphatic compounds being complex and recalcitrant, and carbonyl groups being incompletely decomposed (Gunina & Kuzyakov, 2015; Lorenz et al., 2007; Pérez et al., 2002). Thus, the persistence of soil organic carbon (SOC) relies on the molecular composition of SOM (Yang et al., 2022). The composition of mammal organic inputs, influenced by the diversity of their traits, may have consequences on SOM recalcitrance (resistance to decomposition) and thus on the persistence of SOC (long‐term storage), which highly depends on SOM stabilization through strong organo‐mineral associations, which are known to be less amenable to microbial attack (Feng et al., 2014; Lehmann & Kleber, 2015; Tamrat et al., 2019). Studying SOM composition and recalcitrance improves our understanding of SOC persistence and stabilization and thus provides more accurate predictions of carbon sequestration and climate change impacts (Heckman et al., 2022; Mikutta et al., 2006; Six et al., 2002). As not all forms of carbon are equally resistant to microbial mineralization, understanding the relationships between animal species traits and the type and content of organic matter (OM) accumulated in the soil will help to understand the mechanisms responsible for the causal relationship between mammal diversity and carbon storage in Amazonian soils.

We know that in the northern Amazon biome, mammals contribute to soil carbon concentration through their organic inputs resulting from feeding interactions (Sobral et al., 2017), and influence the type of OM entering decomposition pathways and accumulating in soil. Mammal richness contributes to SOM rich in nitrogen, sulfur, and iron while tree richness leads to SOM rich in carbonyl and aliphatic groups. Thus, mammal and tree diversity can have complementary effects on SOM composition (Losada et al., 2023), but also the diversity of mammals can interact with the composition and diversity of trees (Moorhead et al., 2017; Souza et al., 2022; Villar et al., 2020). Additionally, trait variation within assemblages can also affect carbon acquisition and retention in the organisms, influencing the fluxes and accumulation of carbon in soil (Sobral et al., 2023). For example, mammals differ in their ability to digest complex C‐rich organic compounds (as aliphatic and carbonyl moieties) that later contribute to SOM recalcitrance, reducing OM decomposition and favoring SOC accumulation (Bardgett & Wardle, 2003; Sitters et al., 2017; Stevens & Hume, 2004). Thus, we predict that trait diversity in mammal assemblages may lead to differences in SOM composition with potential impacts on decomposition rates and SOC persistence.

In this study, we aimed to reveal whether functional diversity of species, and not just species richness in mammal assemblages is relevant to variations in SOM composition in northern Amazon soils. First, we characterized the molecular (FTIR‐ATR) and elemental (ED‐XRF and CNH analysis) SOM composition of 401 topsoil samples collected on 83 transects around 13 villages in the Amazon biome (Guyana), for which we also know the abundance and composition of mammal assemblages. Second, we assessed the functional diversity of the 83 mammal assemblages, using species count data from animal surveys and trait data from the open‐access database Amniote (Myhrvold et al., 2015). Third, we determined whether and to what extent there is a relationship between mammal functional diversity and the content and type of SOM accumulated in topsoils. This relationship would suggest that the functional diversity of mammal assemblages could be affecting ecosystem functions such as biogeochemical cycles, nutrient recycling, and soil carbon storage.

## 2 MATERIALS AND METHODS

### 2.1 Study area

Our study area covers 4,800,000 ha of the northern Amazon in the Rupununi region of Guyana (Fragoso et al., 2016; Iwamura et al., 2014; Luzar et al., 2011, 2012; Read et al., 2010; Sobral et al., 2017). It is dominated by tropical forest and savanna systems, and characterized by a low human occupation due to poor access (Luzar et al., 2012). This region harbors an outstanding biodiversity, including 7112 vascular plant species (Funk et al., 2007) and 130 mammal species (Lim & Engstrom, 2005). The climate is tropical humid, with an average annual precipitation of 2375 mm and an average annual temperature of 26°C (Hijmans et al., 2005). The area is predominantly inhabited by Makushi and Wapishana people (Luzar et al., 2012), who traditionally practice hunting, fishing, and farming (Read et al., 2010). Soils are primarily loamy (44% sand and 34% silt) and sandy (91% sand) in the study area, while clay soils (51% clay) are dominant in northern and southern areas of Guyana (Jiménez Espinosa et al., 2022).

### 2.2 Field design

**FIGURE 1:**
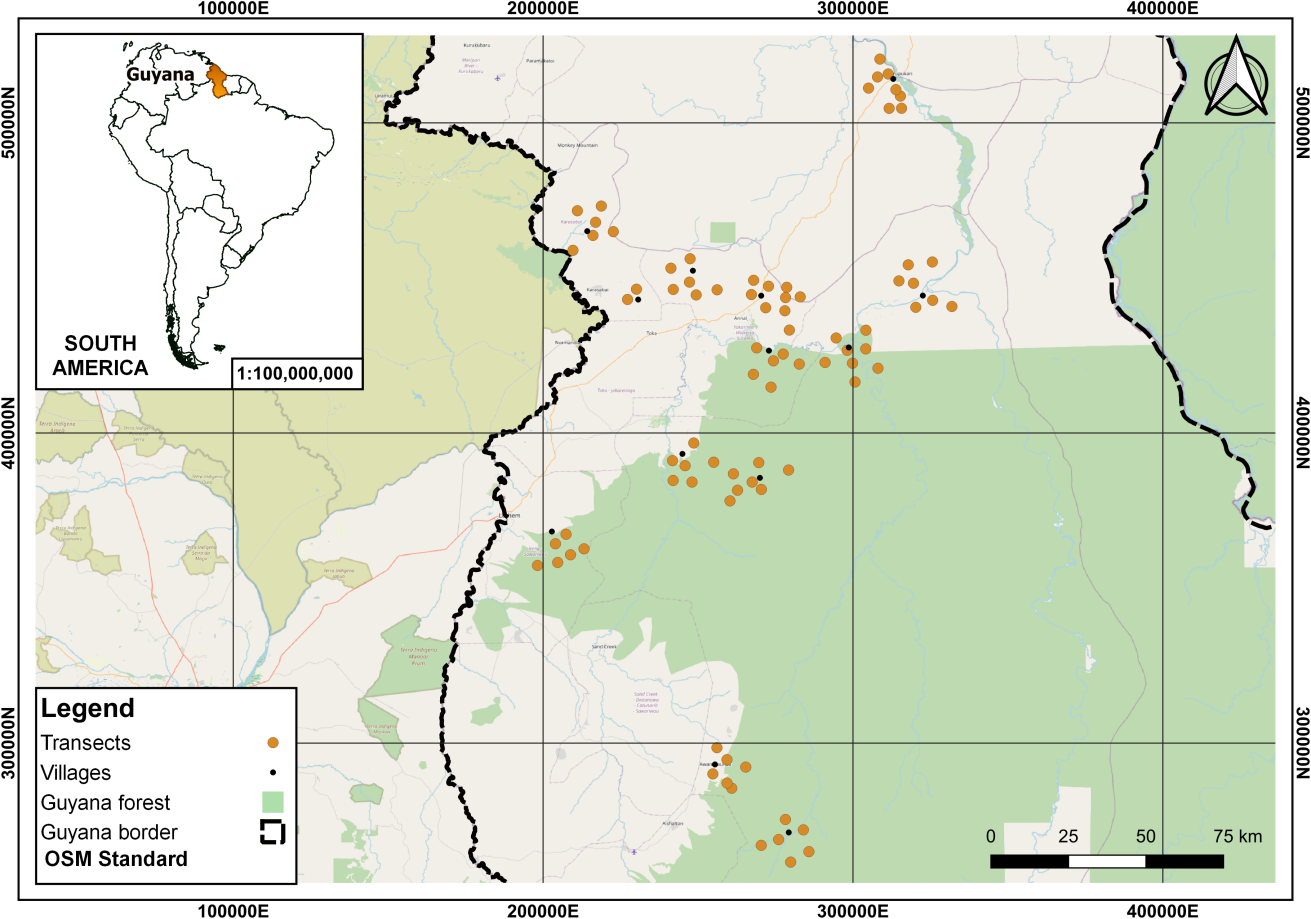
Map of the study design in Guyana (WGS 84/UTM zone 21N), with the geographic location of the 83 transects surveyed for assemblages of mammal and trees and soil characteristics (orange dots), by trained local paratechnicians from 13 villages (black dots) in a forest‐savanna system (layer: https://www.openstreetmap.org, OpenStreetMap©, CC‐BY‐SA), created with QGIS Desktop version 3.22.0 for Windows (QGIS Development Team, 2022).

Four hundred and one soil samples were collected in the top 10 cm (after removal of surface litter) from pits excavated in 8 different plots along 83 linear transects of 4 km length arranged around 13 different local villages (Figure 1). Forty‐eight terrestrial and arboreal mammal species were recorded on the same transects by trained local paratechnicians from the same villages; volant mammals and small terrestrial mammals were not sampled given the limitation on recording species occurrence (Fragoso et al., 2016, 2019; Sobral et al., 2017). Tree species were recorded for 24,552 individuals (DBH > 25 cm, in 2008) on 72 out of the 83 transects (22% of the 163 taxa surveyed were identified to genus level), and all taxa were identified monthly by fruits and seeds on the ground on the 83 transects (2007–2010). We used both data sets to estimate tree species richness from fruit counts in the 83 transects (as Sobral et al., 2017).

### 2.3 Soil organic matter composition

#### 2.3.1 Soil geochemical composition (ED‐XRF and CNH analysis)

The quantitative elemental composition of the 401 topsoil samples (0–10 cm) was analyzed by energy dispersive X‐ray fluorescence (ED‐XRF) spectrometry, using a XEPOS spectrometer (Ametek Corporation). First, each sample was air‐dried, sieved (<2 mm), ground finely (1–3 g), and compressed with a plunger (250–260 gm) on a Teflon cup with a Prolene membrane at the bottom. Four‐point ED‐XRF spectra obtained from each cup were averaged and compared with the values obtained by measuring a certified reference material (NBS‐1572; Gaithersburg MD). We considered the total content of S, Fe, Si, and Al obtained by the ED‐XRF spectrometry to assess soil inorganic components (see Table S1). In addition, C and N concentrations (%) were also determined for all samples previously oven‐dried (110°C), sieved (<2 mm), weighed on a Mettler Toledo microbalance (+0.0005 mg), and then combusted in a Carlo Erba NA‐1500 elemental analyzer (Sobral et al., 2017). The total elemental content of C, N, S, Fe, Si, and Al was centered log‐ratio (CLR) transformed for removing closure effects of analytical chemical data prior to undertaking a Principal Component Analysis (Aitchison, 1984).

#### 2.3.2 Soil spectroscopic signal (FTIR‐ATR)

Topsoil samples were analyzed by Fourier transform infrared (FTIR) spectroscopy with attenuated total reflectance (ATR). FTIR‐ATR spectra were obtained at 4 cm^−1^ resolution in the mid‐infrared (MIR) region of 4000–400 cm^−1^, by averaging 100 scans, using an AGILENT CARY 630 FTIR spectrometer (Agilent Technologies Inc.). The MIR spectral bands for all samples were firstly standardized (“*Z*‐score”) and processed with {*andurinha*} R‐package (Álvarez‐Fernández & Martínez Cortizas, 2020) to obtain the average, standard deviation, and second derivative spectra (used for peak identification). We selected the IR spectral bands reflecting the main chemical constituents of the studied topsoils, in particular regarding the composition of SOM (organic functionalities), but also of the inorganic fraction (i.e., primary minerals and clay minerals). For identifying spectroscopic signals present in the analyzed topsoil samples, we selected a total of 15 IR bands related to SOM (Losada et al., 2023): aromatic and aromatic‐nitrogenated (1550 and 1630 cm^−1^), carbonyl (1700–1720 cm^−1^), and aliphatic (2850 and 2920 cm^−1^) groups, but also to inorganic components probably linked to SOM: iron oxides/silicates (530 cm^−1^), quartz (798 and 777 cm^−1^), kaolinite (911, 3620, and 3694 cm^−1^), or both SOM and inorganic components, i.e., carbohydrates/silicates (1823 and 1838 cm^−1^), since the fractionation of their variance in the PCA (see below) enables discrimination between both soil components.

#### 2.3.3 Principal component analysis (PCA)

The concentrations of six major chemical elements (CLR‐transformed) and peak absorbance of 15 IR bands selected from the 401 topsoil samples from 83 transects were analyzed by PCA using the principal() function of the {*psych*} R‐package (Revelle, 2021). The first three principal components (PC) extracted accounted for 68% of the total variance and the underlying gradients represented by the three components were identified based on the factor loadings (Table S1, modified from Losada et al., 2023). In relationship to SOM composition, the second component, PC2 (21.9% of total variance), was identified as reflecting total SOM content, as it mainly showed large positive loadings for C, N, and S concentrations, and absorbance of aromatic‐nitrogenated (1550 and 1630 cm^−1^) and carboxylated (1700 cm^−1^) SOM. The third component, PC3 (15.3% of total variance), essentially reflected compositional differences in SOM regarding carbonyl content and aliphaticity, represented by large positive loadings of carboxylic/carboxylated (1720–1700 cm^−1^) and aliphatic (2850 and 2920 cm^−1^) functionalities. Note that the first component, PC1 (30.7%), corresponded to an inorganic signal which reflected a gradient of anticovariation of secondary clay minerals content (i.e., kaolinites and iron‐aluminum oxides), versus primary minerals content (i.e., quartz). Although the inorganic composition of the studied topsoils is not the focus of the current study, we included it in our analyses because variations in clays and iron‐aluminum oxides content may affect SOM composition and stabilization processes (Feng et al., 2014). The PC samples' scores were aggregated by the mean at the transect level (*n* = 83) after the standardization of MIR spectra and peak selection with {*andurinha*} routine to preserve variance at the sample level.

### 2.4 Counting surveys

A total of 102,044 individuals of 48 mammal species (41 genera, 23 families, 8 orders; see Table S2) were recorded along 83 transects over 3 years (2007–2010), through monthly sampling by direct observation (*n* = 19,347 individuals) and by animal signs (*n* = 82,697 individuals by burrows, feeding organic remains, tracks, hairs, etc.). This equates to a sampling effort of 4625 mammal surveys conducted by trained local paratechnicians (Fragoso et al., 2016, 2019). We combined mammal species data from both methods to determine mammal species richness for each transect‐level assemblage. We aggregated the sum of mammal species counts at the transect level for a total of 83 mammal assemblages matched with known topsoil geochemical and molecular composition, since functional evenness (FEve) and functional divergence (FDiv) indices account for species relative abundances within each assemblage (Villéger et al., 2008).

### 2.5 Species traits

Mammal trait data were obtained from the open‐access Amniote Life History Database (Myhrvold et al., 2015). This database provides a single value for each trait and species aggregated by the mean (or median) of all values collected from different authors' sources. Only three continuous traits were available for the 48 mammal species surveyed (see Table S2) to calculate the functional diversity — FD (Laliberté et al., 2014; Villéger et al., 2008): body mass (as the body mass of an adult individual in g, see “adult_body_mass_g”: 175–950,000 g; 4.44 ± 2.43 records); offspring number (as the number of offspring born per litter, see “litter_or_clutch_size_n”: 1–10 individuals; 3.40 ± 1.77 records); body length (as the body length of an adult individual in cm, from snout to tail base including head, see “adult_svl_cm”: 20–60.28 cm; 2.08 ± 0.69 records). A potential limitation in this step was that the calculation of some FD indices needs trait data for all species involved in the assemblage so that the number of traits available for all mammal species was compromised. Although there is no limit on the number of traits for FEve calculation, functional richness (FRic) and FDiv indices rely on finding the minimum convex hull that includes all species within a functional assemblage (Villéger et al., 2008).

### 2.6 Functional diversity (FD) indices

**FIGURE 2:**
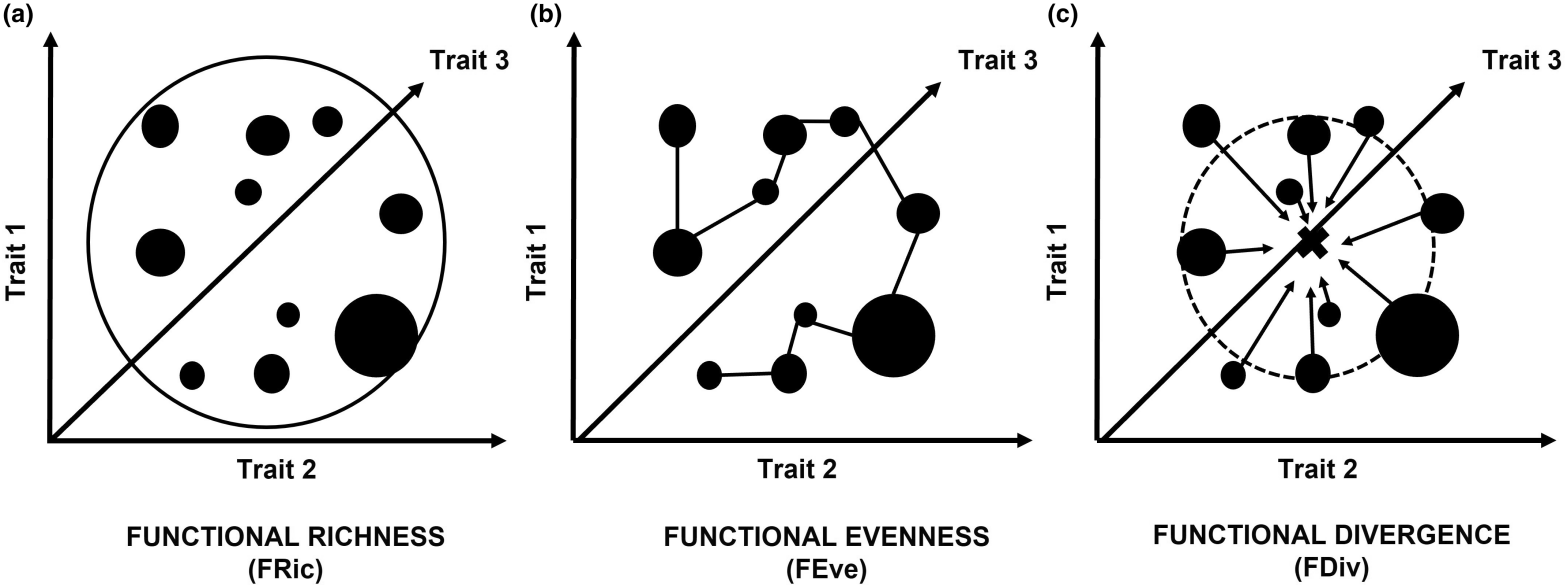
Conceptual explanation of each of the three metrics of Functional Diversity (FD) assessed in our study: (a) Functional Richness (FRic), which measures the volume of the multidimensional FD space occupied by the species traits within an assemblage; (b) Functional Evenness (FEve), which measures the uniformity of the distribution of the species traits of an assemblage within the phenotypical trait space (considering branch distances and abundances of each species); and (c) Functional Divergence (FDiv), which measures the degree of disparity among the species traits of each assemblage within the phenotypical trait space (considering the assemblage measure regarding the centroid of the hypervolume occupied by the species traits of the assemblage).

We calculated three metrics of the multidimensional functional diversity (FD) using the dbFD() function of the {*FD*} R‐package (Laliberté et al., 2014; Laliberté & Legendre, 2010). We used the sum of mammal species counts per transect and the standardized mean values of the species traits obtained in the Amniote Life History Database (Myhrvold et al., 2015), of the 48 mammal species surveyed in all transects. For each mammal assemblage (*n* = 83), the FD space was defined as a multidimensional theoretical space (Convex‐Hull hypervolume) that is delimited by the traits of all the species present within each assemblage, and three metrics of FD were calculated (see Figure 2): the functional richness (FRic), which quantifies the volume of the phenotypical space occupied by the species traits of each assemblage; the functional evenness (FEve), which quantifies the uniformity in the distribution of species traits of each assemblage within the multidimensional FD space (considering species abundances) and is higher when species traits are more uniformly distributed across the phenotypical space; and the functional divergence (FDiv), which quantifies the disparity in the distribution of species traits of each assemblage within the FD space (considering species abundances), being higher when more species have more unusual or extreme traits values relatively to assemblage mean (Villéger et al., 2008). All the traits we included to assess FD for the mammal assemblages are commonly used in mammal functional diversity studies (Flynn et al., 2009; Gorczynski & Beaudrot, 2021; Hempson et al., 2015).

### 2.7 Statistical analysis

We built three independent Linear Mixed Models (LMMs) using the lme() function of the {*nlme*} R‐package (Pinheiro et al., 2023; Pinheiro & Bates, 2000), where the response variables were the three PCs aggregated at the transect level (*n* = 83) with normal distribution and identity link function, and the village was included as a random factor. FD indices were included as fixed effects: the functional richness (FRic), functional evenness (FEve), and functional divergence (FDiv). We also included mammal and tree richness and the respective interaction term into the models to separate the effect of the diversity of mammal traits from the specific richness of both, mammals and trees, and their interaction. To control for the effect of environmental heterogeneity among transects, we also included as fixed effects: the distances to the nearest village, the nearest road, and the nearest river (m), the longitude (X coordinate mean in m) and latitude (Y coordinate mean in m) in the WGS84 UTM 21N zone, the mean annual temperature (°C) and annual precipitation (mm), and the dominant lithology of each transect as a factor defined by eight classes: (i) Continental sands and silts under thin Tertiary cover, (ii) Gabbronorite sills and large dikes, (iii) Granitoids including diorite, Makarapan riebeckite granite, pyroxene granite, (iv) Granulites and charnockites, (v) Greenstone belts mainly intermediate metavolcanics, (vi) High‐grade gneisses, (vii) Subvolcanic granites, and (viii) Acid or intermediate volcanics (Guyana Geology and Mines Commission, 2010). All the continuous variables were standardized (mean = 0; SD = 1) due to model convergence reasons and for comparing the relative effects sizes of continuous predictors. For all analyses, the best minimum adequate model fitted by “ML“ method was selected starting with a saturated model (containing all the cited covariables and the interaction term between mammal and tree richness) and then using the stepAIC() function of the {*MASS*} R‐package (Venables & Ripley, 2002) for (both “backward” and “forward”) stepwise selection to remove the non‐significant terms following the AICc criterium (Burnham & Anderson, 2004) and final models were fitted by “REML” method. For all the models, we previously checked for multi‐collinearity using the vif() function (VIF < 10) of the {*car*} R‐package (Fox & Weisberg, 2019). All statistical analyses were performed in RStudio environment (R Studio Team, 2020) for R version 4.2.1 for Windows (R Core Team, 2021).

## 3 RESULTS

**FIGURE 3:**
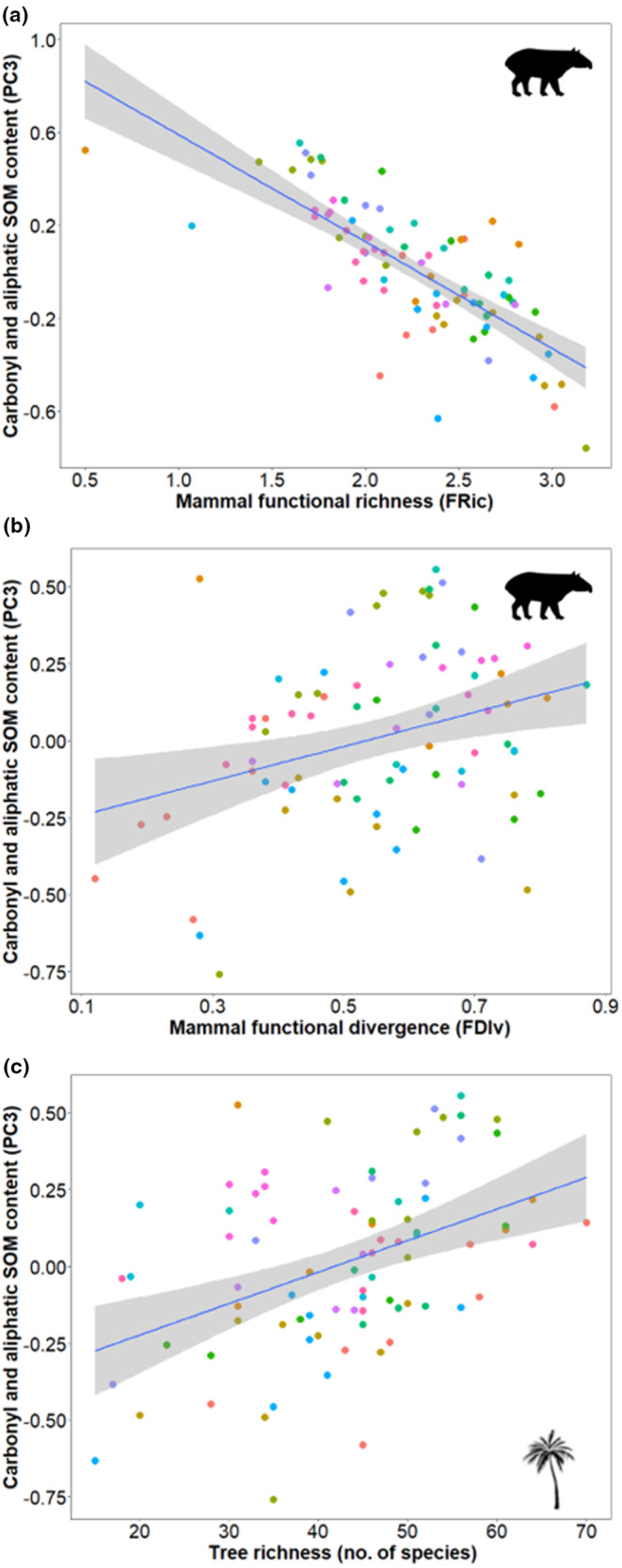
LMM predicted values of the carbonyl and aliphatic SOM content (PC3 scores mean per transect) in response to (a) mammal functional richness (FRic) and (b) functional divergence (FDiv), and (c) tree richness (no. of species) among 83 transects from Amazon biome (Guyana transect arrays around each village represented by different colored‐dots).

Total SOM content (PC2, Table 1b) increased with geographical longitude (towards the west of Guyana) and marginally with distance to the nearest village (towards remote areas). Carbonyl and aliphatic SOM decreased with mammal functional richness (PC3, Table 1c, Figure 3a) and increased with mammal functional divergence (Figure 3b) and with tree‐specific richness (Figure 3c). Thus, mammal trait diversity was more relevant for the content of this SOM composition than mammal‐specific richness. The number of species of tree assemblages has an opposite effect to that of mammal functional richness on PC3 (Table 1c).

**TABLE 1:** Results of Linear Mixed Models (LMMs) after reduction by minimum adequate model performed to analyze the effect of functional diversity measures (FRic, FEve, FDiv) calculated for mammal assemblages on the variation of topsoil geochemical and molecular composition of SOM: (a) PC1, (b) PC2, and (c) PC3 (PC scores mean of topsoil samples aggregated by original transect) among 83 assemblages around 13 different villages (as random) from Amazon biome (Guyana).

Response variable	Effects	Variance	SD	Estimate	SE	df	Wald chi‐square	*p*‐Value
(a) Clays *vs* quartz content (PC1) *n =* 83	**Precipitation**			−0.410	0.094	1	19.193	**<.001**
	**FEve mammals**			−0.195	0.075	1	6.779	**.009**
	Village (random)	0.046	0.215					
(b) Total SOM content (PC2) *n =* 83	*Distance nearest village*			0.107	0.062	1	2.945	*.086*
	**Longitude**			0.386	0.150	1	6.669	**.010**
	Village (random)	0.270	0.520					
(c) Carbonyl and aliphatic SOM content (PC3) *n =* 83	**Tree richness**			0.147	0.067	1	4.841	**.028**
	**FRic mammals**			−0.237	0.067	1	12.587	**<.001**
	**FDiv mammals**			0.135	0.067	1	4.035	**.045**
	Village (random)	2.555E‐09	5.055E‐05					

*Note*: In bold, all significant statistical effects (*p* < .05), and in italics, the marginal effects.

Although the inorganic component in the Amazonian topsoils was not the objective of this study, the LMM of PC1 was reported as well (Table 1a). We detected a negative relationship between mammal functional evenness (FEve) and the variation in the content of clay minerals (kaolinite and iron‐aluminum oxides) versus quartz (PC1): topsoils had more clay minerals in transects where mammal assemblages showed a lower functional evenness (Table 1a).

## 4 DISCUSSION

We previously found that mammal and tree species richness contribute positively to soil carbon concentration (Sobral et al., 2017) and inversely to the content of carbonyl and aliphatic soil organic matter (SOM) in Amazonian topsoils, and that mammal species richness contributes positively to SOM rich in nitrogen, sulfur, and iron (Losada et al., 2023). Now, we find that mammal trait‐diversity metrics such as functional richness (FRic) and functional divergence (FDiv) are inversely related to carbonyl and aliphatic SOM. Lower mammal functional richness but higher functional divergence were related to higher content of carbonyl and aliphatic SOM. Mammal species richness was not retained in the models, suggesting that mammal species traits are better predictors of the variation of carbonyl and aliphatic content in SOM than the number of species in mammal assemblages. In fact, trait‐based measures can often predict better the effects of animal assemblages on ecosystem functioning than the species richness (Gagic et al., 2015), and mammal species richness and functional diversity are decoupled across multiple regions of the world (Oliveira et al., 2016).

Mammal functional diversity is linked to carbon cycling because mammal traits such as body size, body mass, and fecundity relate to slow‐fast and conservative‐acquisitive strategies followed by species, which in turn relate to carbon pools and fluxes in the ecosystem (Capdevila et al., 2020; Junker et al., 2022; Sobral et al., 2023). Our study shows that mammal assemblages with more diverse traits (functional richness) are associated with SOM rich in nitrogen, sulfur, and iron, likely stabilized by strong organo‐metal (Fe) associations that promote long‐term soil organic carbon (SOC) persistence (Feng et al., 2014; Mikutta et al., 2006). Conversely, mammal assemblages with more extreme traits (functional divergence) contribute to recalcitrant SOM rich in carbonyl and aliphatic compounds, which might reduce organic matter (OM) decomposition by microbes in favor of SOC persistence (Lehmann & Kleber, 2015). Thus, combinations of more diverse and disparate traits within mammal assemblages may lead to stabilized and recalcitrant SOM that enhance long‐term SOC persistence (Kristensen et al., 2022; Lehmann & Kleber, 2015) depending on the microbial response to SOM recalcitrance and molecular complexity (Lehmann et al., 2020).

Trophic groups vary in their traits and in the chemical composition of organic inputs that organisms generate through feeding activities and that have different impacts on soil carbon distribution and storage (Andriuzzi & Wall, 2018; Kristensen et al., 2022; Monk & Schmitz, 2022). Body size and mass relate to mammal diet preferences that affect soil nutrient stoichiometry (Hempson et al., 2015; le Roux et al., 2020). Carnivores produce feces with a low C:N ratio (N‐rich OM), promoting a rapid microbial decomposition (Benbow et al., 2019), while herbivores dung shows high C:N ratios (C‐rich OM) which may enhance carbon storage (Kristensen et al., 2022). Large herbivores generate dung with high N:P ratios, potentially limiting soil nutrient availability for plants (le Roux et al., 2020). In our study system, mammal assemblages are composed of ungulates, primates, rodents, and carnivores that vary in body mass, and across transects, the biomass of herbivores exceeds that of carnivores (Table S2, Fragoso et al., 2016). Dominant herbivore biomass may lead to long‐term SOC persistence (Kristensen et al., 2022; Naidu et al., 2022; Schmitz & Leroux, 2020), by accumulating more recalcitrant SOM, rich in carbonyl and aliphatic compounds, as accumulated in mammal assemblages with more disparate traits. Besides, animal remains from carnivory and frugivory are important contributors to soil carbon in our study system (Sobral et al., 2017), which might link to the N‐rich SOM accumulated by mammal assemblages with more diverse traits (Peziol et al., 2023; Villar et al., 2021). Thus, mammal functional richness and divergence might have the opposite effect on SOM recalcitrance in our system, with potential impacts on microbial decomposition and SOC persistence (Lehmann et al., 2020; Lehmann & Kleber, 2015).

Our molecular analysis evaluates the quantity and SOM rich in carbon (carbohydrates, aliphatics, carbonyls, and aromatics) or nitrogen (amides from proteins), with distinct implications for SOM recalcitrance (resistance to degradation by microbes). Polysaccharides, mainly from plant tissues, contribute to the labile SOM, which is easily biodegradable (Kögel‐Knabner, 2002). Aliphatics, carbonyls, and aromatics, which tend to accumulate as a result of microbial degradation, contribute to recalcitrant SOM, which resists degradation by microbes (Six et al., 2001). Moreover, elemental composition (C, N, S, Fe, Si, Al) was integrated into the PCA to improve our interpretation of molecular SOM composition (see Table S1, Losada et al., 2023). Carbon is the main constituent of SOM while nitrogen and sulfur are the main elements of mineralized SOM in tropical soils, that influence SOC accumulation (Tipping et al., 2016). Iron, aluminum, and silicon also concentrate in tropical soils due to intense weathering: Si is the basis of resistant primary minerals, such as quartz, while Fe and Al are present in secondary minerals, such as clays and oxi‐hydroxides that contribute to SOC stabilization (Souza et al., 2017). Given that all these SOM components can occur simultaneously and interact with each other, it is important to understand their relationships together if we aim to understand the complexity of soil carbon dynamics in nature.

In this investigation, we did not study functional diversity of tree assemblages because of a lack of species‐level traits for the whole assemblage, but we found that tree richness is linked to recalcitrant organic components, such as aliphatic compounds, which are less easily and less efficiently degraded by soil microbial communities (Cotrufo et al., 2013) and also poorly digested by large herbivores (Freeland & Janzen, 1974; Jung & Allen, 1995). Our work shows that carbonyl and aliphatic SOM seems to be enhanced in areas with high tree richness and high mammal functional divergence, in comparison to more labile compounds (e.g., polysaccharides). Thus, different accumulation patterns linked to mammal and tree assemblages and the traits of different species within mammal assemblages can explain the inverse effect detected between mammal functional richness and tree richness on the content of carbonyl and aliphatic SOM. This is consistent with our previous findings on the complementary effects of mammal and tree richness on SOM composition: mammal richness is related to SOM rich in N, S, and Fe, while tree richness is related to SOM rich in carbonyl and aliphatic compounds (Losada et al., 2023).

Overall, SOM content is positively related to environmental factors, such as longitude and remoteness (marginal effect of distance to the nearest village). Different underlying processes that follow a longitudinal gradient and are linked to human presence, such as restricted vertebrate movements due to human settlements (Doherty et al., 2021), or the hunting habits of local communities already reported in this region (Iwamura et al., 2014; Luzar et al., 2011; Read et al., 2010), could limit the quantity of vertebrate carcasses accumulated at a site (Benítez‐López et al., 2019; Morant et al., 2023; Roopsind et al., 2017). Less deposition of carcasses on the soil may result in a slower rate of SOM decomposition by microbes and reduced nitrogen inputs (MacDonald et al., 2014; Quaggiotto et al., 2019). A reduction in microbial decomposition rates might result in a decrease in the total amount of SOM (Carter et al., 2007), which may be dominated by aliphatic and carbonyl compounds that contribute to SOM recalcitrance (Six et al., 2001).

Finally, we observed that the clay fraction of the studied topsoils negatively covaried with the functional evenness of mammal assemblages. Topsoil clay content (PC1) was higher in areas with lower values of mammal functional evenness, i.e., lower uniformity in the distribution of species traits of mammal assemblages within the phenotypical space. Given the prior positive relationship found between the tree richness and clay content for the studied topsoils (Losada et al., 2023), if mammal assemblages with lower trait evenness were associated with more diverse tree assemblages, they might indirectly promote clay formation/iron‐aluminum oxides accumulation in these soils. These clay minerals with iron/aluminum oxides can potentially stabilize SOM as accumulated by more diverse mammals through organo‐metal(Fe) associations, which are known to be less susceptible to microbial attack (Feng et al., 2014; Singh et al., 2018). Alternatively, habitat variables such as soil mineralogy and vegetation composition could influence habitat selection by mammals, mammal assemblage composition, and therefore trait diversity. Soil nutrient availability in tropical forests plays a role in trophic cascades, influencing mammal biomass through changes in the diversity and chemical composition of trees (Peres, 2008). However, we note that mammal functional diversity in the tropics is primarily influenced by habitat productivity and anthropogenic disturbances rather than other factors such as tree size, leaf litter, and soil clay content (Boron et al., 2019; de Oliveira et al., 2019; Gorczynski et al., 2021).

In summary, our work shows that mammals are key to the cycling of carbon in the Amazon, producing effects additional to those of tree assemblages and that the trait composition of mammal assemblages might be more important than their richness when predicting the type of soil organic matter accumulated. Evidence is rising that richness and trait composition of mammal assemblages are key to carbon cycling and storage in soils.

## AUTHOR CONTRIBUTIONS

**María Losada:** Data curation (equal); formal analysis (equal); investigation (equal); methodology (equal); visualization (lead); writing – original draft (lead); writing – review and editing (lead). **Mar Sobral:** Conceptualization (equal); data curation (equal); formal analysis (equal); funding acquisition (equal); investigation (equal); methodology (equal); supervision (lead); writing – original draft (lead); writing – review and editing (lead). **Kirsten M. Silvius:** Funding acquisition (equal); investigation (equal); methodology (equal); writing – review and editing (equal). **Sara Varela:** Formal analysis (equal); funding acquisition (equal); investigation (equal); supervision (equal); writing – review and editing (equal). **Antonio M. Martínez Cortizas:** Conceptualization (equal); formal analysis (equal); funding acquisition (equal); investigation (equal); methodology (equal); supervision (equal); writing – review and editing (equal). **José M. V. Fragoso:** Funding acquisition (equal); investigation (equal); methodology (equal); writing – review and editing (equal).

## CONFLICT OF INTEREST STATEMENT

The authors declare no conflict of interest.

## Supporting information


Table S1

## Data Availability

Due to human rights concerns, and based on best practices for Free Prior Informed Consent (FPIC), the CARE Principles for Indigenous Data Governance, and respect for indigenous intellectual property rights, an agreement exists between the co‐senior author and principal investigator of the project in the context of which biodiversity data and soil samples were collected (Project Fauna, co‐senior author, J.M.V.F.), and the project's indigenous collaborators, that no raw or derived data can be publicly shared that could identify either communities or individuals; that raw and derived data use is restricted to publications and other uses that will not injure the territories, livelihoods, beliefs or intellectual property rights of the indigenous communities. The original code generated in this paper is available from the corresponding author upon request. Requests for additional information required to reanalyze the data reported in this paper should be addressed to José M.V. Fragoso (fragoso1@mac.com) to determine whether the data can be made available on a case‐by‐case basis without identifiers.
