# Correction to “Mammal traits and soil biogeochemistry: Functional diversity relates to composition of soil organic matter”

**DOI:** 10.1002/ece3.10543

**Published:** 2023-09-15

Losada, M., Sobral, M., Silvius, K. M., Varela, S., Martínez Cortizas, A. M., & Fragoso, J. M. V. (2023). Mammal traits and soil biogeochemistry: Functional diversity relates to composition of soil organic matter. Ecology and Evolution, 13, e10392. https://doi.org/10.1002/ece3.10392

In the correspondence data of the “Authors' list” section, the email address “sobral.bernal.mar@gmail.com” was incorrect. This should have read: “mar.sobral.bernal@usc.es”.

We apologize for this error.

