# Supplementary material for: Mammal traits and soil biogeochemistry: Functional diversity relates to composition of soil organic matter
Source: Ecol Evol. 2023 Aug 17;13(8):e10392. doi: 10.1002/ece3.10392 (PMC10433116; doi:10.1002/ece3.10392)
Supplement: Supplementary file 1 — Table S1 [file ECE3-13-e10392-s001.docx]

**Supporting Information**

**Table S1.** Factor loadings of the 3 first PCs extracted from PCA of topsoil geochemical composition (total content of C, N, S, Si, Al, Fe with CLR transformation) and molecular composition (standardized absorbance of 15 IR bands selected as main inorganic and organic components) variables, and which explained a 67.9% of total variance (% explained by each PC in table) among 401 topsoil samples from 83 transects in Guyana.

| **Soil composition** | **PC1^a^**  **(30.7%)** | **PC2^b^**  **(21.9%)** | **PC3^c^**  **(15.3%)** |
| --- | --- | --- | --- |
| silicon | **-0.38** | **0.5** | **-0.36** |
| aluminum | **0.32** | **0.67** | **-0.38** |
| iron | **0.69** | **0.37** | -0.25 |
| iron oxides/silicates | **0.93** | 0.11 | -0.02 |
| clay (kaolinite) | **0.89** | 0.1 | -0.04 |
| clay (kaolinite) | **0.89** | 0.06 | -0.12 |
| clay (kaolinite) | **0.91** | 0.07 | -0.06 |
| quartz | **-0.94** | -0.15 | -0.07 |
| quartz | **-0.92** | -0.13 | -0.03 |
| carbon | -0.07 | **0.89** | -0.23 |
| nitrogen | 0.07 | **0.86** | -0.29 |
| sulphur | **-0.36** | **0.8** | **-0.31** |
| aromatic SOM | -0.13 | **0.6** | **0.36** |
| aromatic-N SOM | -0.11 | **0.63** | 0.29 |
| carbonyl SOM (carboxylates) | -0.01 | **0.36** | **0.71** |
| carbonyl SOM (organic acids) | 0.02 | 0.26 | **0.69** |
| carbonyl SOM (carboxylic acids) | 0.14 | 0.17 | **0.72** |
| aliphatic SOM | -0.17 | **0.35** | **0.65** |
| aliphatic SOM | -0.1 | **0.43** | **0.54** |
| carbohydrates/silicates | **0.52** | **-0.31** | **0.30** |
| carbohydrates/silicates | **0.49** | **-0.34** | **0.31** |

**Footnote1.** Each PC represents different gradients in soil components, as follows:

^a^ Clays *vs* quartz content (anticovariation of kaolinite + iron-aluminum oxides versus quartz)

^b^ Total SOM content (C, N, S concentrations + aromatic-nitrogenated and aliphatic SOM)

^c^ Carbonyl and aliphatic SOM content (carbonyl groups + aliphaticity)

| **Table S2.** Mammal species list. | | | |
| --- | --- | --- | --- |
| ***Species***  **Order – Family^[[1]](#footnote-1)^** | **Common name (weight; body length) and other traits^[[2]](#footnote-2),^ ^[[3]](#footnote-3)^**  **Habitat, ecology, and diet ^1, 2^** | **IUCN**  **Red List^1^** | **Photo** |
| *Alouatta seniculus*  Primates – Atelidae | Linnaeus’ red howler (6.4kg; 0.74m), reddish fur with prehensile tail. Emit loud howls. Arboreal, social (2-3) and herbivorous. | 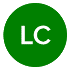 | [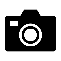](https://search.creativecommons.org/photos/68b634ce-b9c3-487d-ab2d-ed61244e3b9c) |
| *Ateles paniscus*  Primates – Atelidae | Guianan spider monkey (7.8kg; 0.6m), black fur. Diurnal, social (30) and omnivorous. | 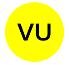 | [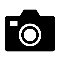](https://search.creativecommons.org/photos/a9670554-dfea-4788-be44-b4a6b5424735) |
| *Bassaricyon gabbii*  Carnivora – Procyonidae | Northern olingo (1.2kg; 0.4m), with reddish fur and long tail. Arboreal, nocturnal, solitary, and omnivorous. | 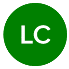 | [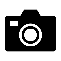](https://search.creativecommons.org/photos/6acabcae-af73-4783-bf52-f78fce1d45ad) |
| *Bradypus tridactylus*  Pilosa – Bradypodidae | Pale-throated three-toed sloth (4kg; 0.5m), dark fur, males with a yellow dorsal spot. Diurnal, solitary and herbivorous. | 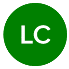 | [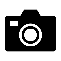](https://search.creativecommons.org/photos/ffa2bc54-fb37-485c-a44e-46cc9a1aaf57) |
| *Cabassous unicinctus*  Cingulata – Clamyphoridae | Southern naked-tailed armadillo (3.9kg; 0.45m), hard carapace and long, sticky tongue. Nocturnal, solitary, and insectivorous. | 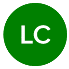 | [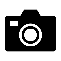](https://search.creativecommons.org/photos/028ff5d8-497b-4b44-8fb7-9f070d38d793) |
| *Cebus apella**  Primates – Cebidae | Black-capped capuchin (2.6kg; 0.49m), brown hair, black at the crown. Prehensile tail. Arboreal, diurnal, social (6-30), and omnivorous (*currently: *Sapajus apella*) | 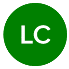 | [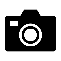](https://search.creativecommons.org/photos/81965612-ea50-489a-bb7d-bc0b45bb1d25) |
| *Cebus olivaceus*  Primates – Cebidae | Guianan weeper capuchin (2.5kg; 0.48m), brown coat, black crown, and light face. Prehensile tail. Arboreal, diurnal, social (12) and omnivorous. | 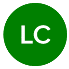 | [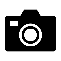](https://search.creativecommons.org/photos/a9166876-3983-4d8c-90ec-341c625ef414) |
| *Cerdocyon thous*  Carnivora – Canidae | Crab-eating fox (5.7kg; 0.67m), short, strong legs and variable reddish-brown fur. Nocturnal, monogamous, and carnivorous. | 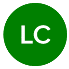 | [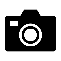](https://search.creativecommons.org/photos/3de33483-27c5-415f-a889-2dee6935b179) |
| *Chiropotes satanas*  Primates – Pitheciidae | Black bearded saki (8.6kg; 0.63m) black hair, beards, and shaggy tail. Diurnal, social (groups of 40) and omnivorous. | 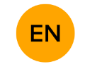 | [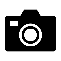](https://search.creativecommons.org/photos/79d3c444-43c2-4407-bff3-e1a0a6383dbf) |
| *Choloepus didactylus*  Pilosa – Megalonychidae | Linnaeus’ two-toed sloth (2.3kg; 0.8m), brown or greenish fur (mutualism with algae), and two strong claws. Arboreal, diurnal, solitary, and herbivorous. | 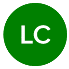 | [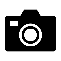](https://search.creativecommons.org/photos/71c1d014-dee0-4a54-8e58-79ae64107c38) |
| *Coendou melanurus*  Rodentia – Erethizontidae | Black-tailed hairy dwarf porcupine (1.4kg; 0.5m) covered with strong grayish quills. Arboreal, nocturnal, solitary, and herbivorous. | 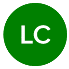 | [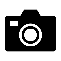](https://www.inaturalist.org/photos/4578998) |
| *Coendou prehensilis*  Rodentia – Erethizontidae | Brazilian porcupine (3.9kg; 0.5m), covered with gray quills, prehensile tail. Arboreal, nocturnal, solitary, and herbivorous. | 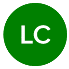 | [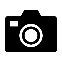](https://search.creativecommons.org/photos/217d5899-7e40-4970-a2e8-228d65264e1a) |
| *Cuniculus paca*  Rodentia – Cuniculidae | Agouti (9kg; 0.6m), reddish-brown fur with white spots on the sides. Living in forests near water, nocturnal and herbivorous. | 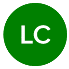 | [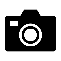](https://search.creativecommons.org/photos/9965ab48-108d-4d4c-ae13-c5a5f55d49e4) |
| *Dasyprocta leporina*  Rodentia – Dasyproctidae | Red-rumped agouti (3kg; 0.6m), reddish fur and short, naked tail. Living in forests, diurnal and herbivorous. | 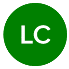 | [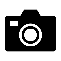](https://search.creativecommons.org/photos/1ce9e7a2-ba05-48c8-bf2f-6d98c1e4b8ab) |
| *Dasypus kappleri*  Cingulata – Dasypodidae | Greater long-nosed armadillo (9.9kg; 0.6m), gray bony carapace and elongated snout. Living in forests, diurnal, solitary and omnivorous. | 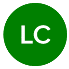 | [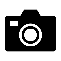](https://search.creativecommons.org/photos/6734cdf8-43ee-40ce-8344-473201a8cf4f) |
| *Dasypus novemcinctus* Cingulata – Dasypodidae | Nine-banded armadillo (4kg; 0.5m), bony carapace, elongated tail and snout. Wide range of habitats, nocturnal and omnivorous. | 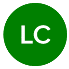 | [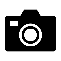](https://wordpress.org/openverse/photos/7fdf678e-19cd-4a33-948d-39d28826e2f7) |
| *Didelphis marsupialis*  Didelphimorphia –Didelphidae | Common opossum (1kg; 0.4m), gray fur with a black mask. Terrestrial-arboreal, nocturnal, solitary, and omnivorous. | 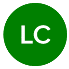 | [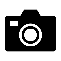](https://search.creativecommons.org/photos/7bfccd63-0c09-410a-b2f9-81710ade5a9d) |
| *Eira barbara*  Carnivora – Mustelidae | Tayra (4.1kg; 0.6m), dark fur, light head. Living in forests, terrestrial-arboreal, diurnal-crepuscular, and omnivorous. | 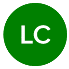 | [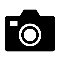](https://search.creativecommons.org/photos/8ed6797f-5f2f-4348-bb54-f506fa7e5080) |
| *Galictis vittata*  Carnivora – Mustelidae | Greater grison (2.3 kg; 0.5 m), slender and elongated body, with short limbs. Terrestrial, crepuscular, and omnivorous. | 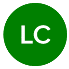 | [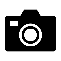](https://search.creativecommons.org/photos/5c6d9db3-e8d9-47fa-aae1-59bca7c71c32) |
| *Herpailurus yagouaroundi*  Carnivora – Felidae | Jaguarundi (7kg; 0.7m) slender body with short legs, gray or reddish fur without spots. Terrestrial, diurnal, solitary, and carnivorous. | 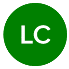 | [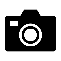](https://search.creativecommons.org/photos/db1702d8-e442-4667-9572-835979aa299d) |
| *Hydrochoerus hydrochaeris*  Rodentia – Caviidae | Capybara (51kg; 1.2m), robust body with brown fur. Living in riparian forests in groups (8-10), diurnal and herbivorous. | 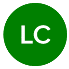 | [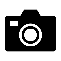](https://search.creativecommons.org/photos/505d7ed7-c82c-478c-a33e-ccf1031ea521) |
| *Leopardus pardalis*  Carnivora – Felidae | Ocelot (10.5kg; 0.9m), yellow fur with brown-black spots. Terrestrial, nocturnal-crepuscular, solitary, and carnivorous. | 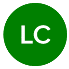 | [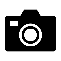](https://search.creativecommons.org/photos/39ac308a-5f9f-4a62-a909-6d179fa6c98a) |
| *Leopardus tigrinus*  Carnivora – Felidae | Northern tiger cat (2.3kg; 0.5m), brown fur with dark spots. Living in forests, terrestrial, nocturnal-crepuscular, solitary, and carnivorous. | 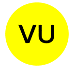 | [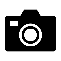](https://wordpress.org/openverse/photos/ca8ddd0b-2024-475c-83ce-82ee5955b950) |
| *Leopardus wiedii*  Carnivora – Felidae | Margay (3.6kg; 0.7m), yellow fur with dark spots. Living in forests, terrestrial, nocturnal-crepuscular, solitary, and carnivorous. | 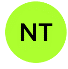 | [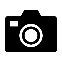](https://search.creativecommons.org/photos/a384f222-6f75-42da-800e-6ed8eb90c767) |
| *Lontra longicaudis*  Carnivora – Mustelidae | Neotropical otter (7kg; 0.7m), elongated, with brown waterproof fur and long tail. Living in riversides, terrestrial-aquatic, solitary and carnivorous. | 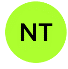 | [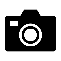](https://search.creativecommons.org/photos/73585402-53f5-4a10-a0cc-1a8378a8ada6) |
| *Mazama americana*  Artiodactyla – Cervidae | American red brocket (23.0kg; 1.3m), reddish fur, males with short and straight antlers. Living in forests, nocturnal, solitary, and herbivorous. | 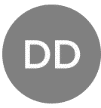 | [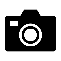](https://search.creativecommons.org/photos/3e6d6ed7-0a45-4752-88a0-2cc0a3b0c83b) |
| *Mazama gouazoubira*  Artiodactyla – Cervidae | Gray brocket (16.6kg; 1.1m), grayish fur, juveniles with white spots and males with unbranched antlers. Nocturnal, solitary, and herbivorous. | 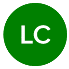 | [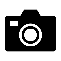](https://search.creativecommons.org/photos/11a83224-3b33-48da-91db-51d82c99cfdb) |
| *Mesomys hispidus*  Rodentia – Echimyidae | Ferreira's spiny tree rat (0.2kg; 0.2m), brown fur. Living in forests, nocturnal, solitary, and omnivorous. | 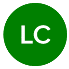 | [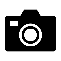](https://search.creativecommons.org/photos/909b80b2-274b-4f23-b2d6-61ab2c18e099) |
| *Myoprocta acouchy*  Rodentia – Dasyproctidae | Red acouchi (0.84kg; 0.4m), reddish-brown fur with a lighter head. Living in forests in groups (7), diurnal and herbivorous. | 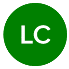 | [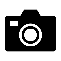](https://search.creativecommons.org/photos/c77a903b-3de9-4b3c-af81-1fa4f8089b5d) |
| *Myrmecophaga tridactyla*  Pilosa – Myrmecophagidae | Giant anteater (30kg; 1.5m), gray and black fur from chest to back. Long trunk with extensible and sticky tongue. Diurnal, solitary and insectivorous. | 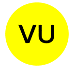 | [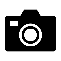](https://search.creativecommons.org/photos/e7ec4695-e72d-4957-9b76-e173588e9643) |
| *Nasua nasua*  Carnivora – Procyonidae | South American coati (4kg; 0.6m), brown fur, cream rings on the tail. Inhabits forests. Terrestrial-arboreal, diurnal, solitary, and omnivorous. | 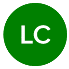 | [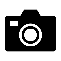](https://search.creativecommons.org/photos/8581f814-7449-4a78-8e2f-adbb99fd24e5) |
| *Odocoileus virginianus*  Artiodactyla - Cervidae | White-tailed deer (65.3kg; 0.2m), grey fur with white spots on face and tail. Males with antlers. Crepuscular, solitary, and herbivorous. | 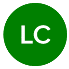 | [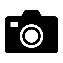](https://search.creativecommons.org/photos/ce4e6a0f-5e16-4b8d-a54e-0f2cca043335) |
| *Panthera onca*  Carnivora – Felidae | Jaguar (81.1kg; 0.6m), yellow fur with black spots. Crepuscular, solitary, and carnivorous. | 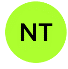 | [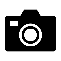](https://search.creativecommons.org/photos/5fe3eef4-0f55-4112-9473-796fb9652835) |
| *Philander opossum*  Didelphimorphia – Didelphidae | Gray four-eyed opossum (0.5kg; 0.3m), grey fur, a white mole over the eyes, long and prehensile tail. Arboreal-terrestrial, nocturnal, and omnivorous. | 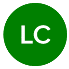 | [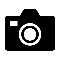](https://search.creativecommons.org/photos/14a1a6e7-9d11-4b5f-bd4c-2783f1182bd7) |
| *Pithecia pithecia*  Primates – Pitheciidae | White-faced saki (1.5kg; 0.5m), black males with white face with grey strands. Living in groups (2-9) and omnivorous. | 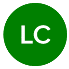 | [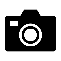](https://search.creativecommons.org/photos/784201c1-210b-4799-b7d9-1804746c481c) |
| *Potos flavus*  Carnívora – Procyonidae | Kinkajou (2.4kg; 0.6m), brown fur, long and prehensile tail. In forests. Arboreal, nocturnal, solitary, and omnivorous. | 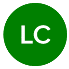 | [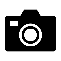](https://search.creativecommons.org/photos/ed54a45d-3047-4395-a85a-7a5009b9c807) |
| *Priodontes maximus*  Cingulata – Clamyphoridae | Giant armadillo (45kg; 1m), with a bony carapace and a large front claw. Nocturnal, terrestrial, solitary, and insectivorous. | 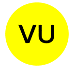 | [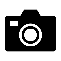](https://search.creativecommons.org/photos/aeffeb3e-884a-45e2-be63-fc9f2bfa12f6) |
| *Procyon cancrivorus*  Carnivora – Procyonidae | Crab-eating raccoon (6.3kg; 0.6m), brown fur, with a black mask. Living in riparian forests, solitary and omnivorous. | 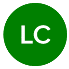 | [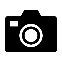](https://wordpress.org/openverse/photos/7740c1b7-cc8b-421d-8172-6c6276b96325) |
| *Pteronura brasiliensis*  Carnivora – Mustelidae | Giant otter (24kg; 1.3m), dark brown fur with white spots on throat and tail. Lives in groups (10-15). Carnivorous. | 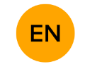 | [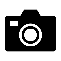](https://search.creativecommons.org/photos/f5ded34f-0818-4ea3-958c-cb5f42796d5a) |
| *Puma concolor*  Carnivora – Felidae | Puma (48kg; 1.7m), yellow-brown fur. Nocturnal, solitary, and carnivorous. | 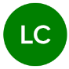 | [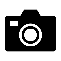](https://wordpress.org/openverse/photos/fe05f525-20bc-4d50-b7f5-877a59602838) |
| *Saguinus midas*  Primates – Callitrichidae | Golden-handed tamarin (0.5kg; 0.3m), black fur with yellow feet. In groups (4-15). Arboreal, diurnal, and omnivorous. | 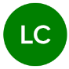 | [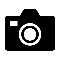](https://search.creativecommons.org/photos/56edbb3c-8937-4b3a-b7bf-82e0178ad737) |
| *Saimiri sciureus*  Primates – Cebidae | Guianan squirrel monkey (0.8kg; 0.3m), short gray fur with yellow legs and a non-prehensile tail. Living in groups of 300, and omnivorous. | 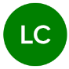 | [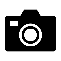](https://search.creativecommons.org/photos/460767b8-486a-4884-aadc-19f1fbf3e7d2) |
| *Sciurus aestuans*  Rodentia – Sciuridae | Guianan squirrel (0.2kg; 0.2m), reddish-gray fur and long, shaggy tail. Diurnal, solitary and omnivorous. | 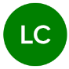 | [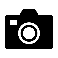](https://wordpress.org/openverse/photos/08d1c982-2345-4b5e-9993-2a6176824ff7) |
| *Speothos venaticus*  Carnivora – Canidae | Bush dog (6kg; 0.7m), brown fur, lighter on the head and with a small tail. Diurnal and carnivorous. | 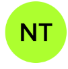 | [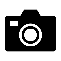](https://search.creativecommons.org/photos/aacf29f6-b794-4e8e-a6be-c3803a4297b0) |
| *Tamandua tetradactyla*  Pilosa – Myrmecophagidae | Southern tamandua (4.5kg; 0.7m), yellow and black fur, prehensile tail, and long trunk. Nocturnal, solitary, and insectivorous. | 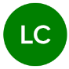 | [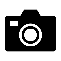](https://search.creativecommons.org/photos/e2d959cd-80c8-41cb-8a49-a0674ae249fe) |
| *Tapirus terrestris*  Perissodactyla – Tapiridae | Lowland tapir (225kg; 2.2m), short grayish-brown fur with an elongated mane and snout. Terrestrial, nocturnal, solitary, and herbivorous. | 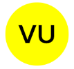 | [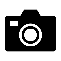](https://search.creativecommons.org/photos/1ded07ba-777b-4652-b7e8-75547bd56492) |
| *Tayassu pecari*  Artiodactyla – Tayassuidae | White-lipped peccary (32kg; 1m), dark fur, white in mouth and cheeks; long snout, large and sharp canines. Terrestrial, diurnal, social (to herds of 400) and herbivorous. | 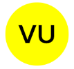 | [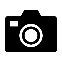](https://search.creativecommons.org/photos/743ea4f8-6afa-4024-beb9-3cef2e55c1a0) |
| *Tayassu tajacu*  Artiodactyla – Tayassuidae | Collared peccary (20.5kg; 1m), dark fur with a light fur collar. Diurnal-crepuscular, social (herds of 6-30), and omnivorous. | 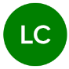 | [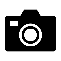](https://search.creativecommons.org/photos/f8cccfaa-d5c9-4033-bed3-479c13b47ab2) |

1. IUCN: The IUCN Red List of Threatened Species, Version 2021-3 [online] Available from: https://www.iucnredlist.org (Accessed 20 January 2023), 2023. [↑](#footnote-ref-1)
2. Marín Brañas, M. and Choclote, J.: Amzonía: Guía ilustrada de flora y fauna, edited by M. Martín Brañas, Serigrafica Industrial S.A, Iquitos, Peru. [online] Available from: https://bibliotecadigital.aecid.es/bibliodig/pub_aecid/es/consulta/registro.do?id=10492 (Accessed 7 March 2022), 2009. [↑](#footnote-ref-2)
3. Myers, P., Espinosa, R., Parr, C. S., Jones, T., Hammond, G. S. and Dewey, T. A.: The Animal Diversity Web, [online] Available from: http://animaldiversity.org (Accessed 20 January 2022), 2006. [↑](#footnote-ref-3)
